# Beyond the Surface: Chronic Lymphocytic Leukemia Diagnosis During Mohs Micrographic Surgery

**DOI:** 10.7759/cureus.66771

**Published:** 2024-08-13

**Authors:** Jessica Forbes Kaprive, Jonmichael Samsel, Alexandra Loperfito, Mallory Towe, Craig Garofola

**Affiliations:** 1 Dermatology, LewisGale Hospital Montgomery, Blacksburg, USA; 2 Dermatology, Edward Via College of Osteopathic Medicine, Blacksburg, USA; 3 Dermatology, Nova Southeastern University Dr. Kiran C. Patel College of Osteopathic Medicine, Fort Lauderdale, USA; 4 Dermatology, River Ridge Dermatology, Blacksburg, USA

**Keywords:** skin cancer, basal cell carcinoma, squamous cell carcinoma, mohs micrographic surgery, chronic lymphocytic leukemia

## Abstract

Chronic lymphocytic leukemia (CLL) is the most common type of leukemia in adults, characterized by the accumulation of abnormal lymphocytes in the blood and bone marrow. Its incidence increases with age, typically affecting older adults, with a median age at diagnosis around 70 years. CLL prevalence varies geographically, with higher rates observed in Western countries compared to Asian populations. Despite advancements in treatment, CLL remains an incurable disease, often managed through monitoring and therapy to control symptoms and slow disease progression. The purpose of this case report is to highlight two unique incidents of previously undiagnosed CLL, incidentally found during Mohs micrographic surgery (MMS). One case features a cutaneous squamous cell carcinoma in situ and the other a basal cell carcinoma. We present these cases to highlight the importance of diagnostic vigilance during Mohs histopathological processing. Diagnosis of CLL is typically through routine complete blood panels. However, these cases present unique initial presentations that warrant careful detection in medical practice. Detecting CLL during the examination of pathology samples from MMS excision may not be common practice, but its presence emphasizes the significance of thorough patient evaluation during medical procedures. This unexpected finding underscores the importance of thorough pathology examination during surgical procedures, highlighting the potential for detecting concurrent or underlying systemic conditions. Early identification of CLL in this context allows for prompt intervention and comprehensive management, emphasizing the necessity of integrated care approaches in medical practice.

## Introduction

Chronic lymphocytic leukemia (CLL) is the most common leukemia found in the United States today [1] The majority of patients with CLL are over the age of 50 years, Caucasian, and asymptomatic at the time of diagnosis [2,3]. Manifestations of this cancer vary greatly, and unique dermatologic manifestations exist including cutaneous malignancy, drug eruptions, varicella-zoster, and graft-versus-host disease presenting later in the disease course [4,5]. Squamous cell carcinoma (SCC) and basal cell carcinoma (BCC) are the most common skin manifestations of CLL, but the association of BCC as the presenting feature is considerably rare [4,5].

We present two distinct cases of an elderly asymptomatic patient who was initially diagnosed with SS in situ (SCCIS) on the superior forehead and a 73-year-old male initially diagnosed with an infiltrative BCC on the inferior back. Both patients were later diagnosed with CLL during the subsequent stages of Mohs micrographic surgery (MMS).

## Case presentation

Case 1

**Figure 1:**
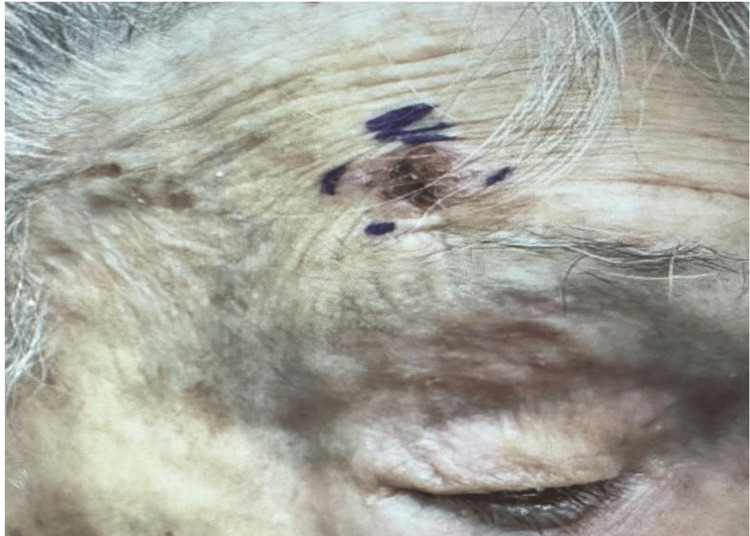
Erythematous crusted plaque on the superior forehead at the presentation that was biopsied via the shave method.

**Figure 2:**
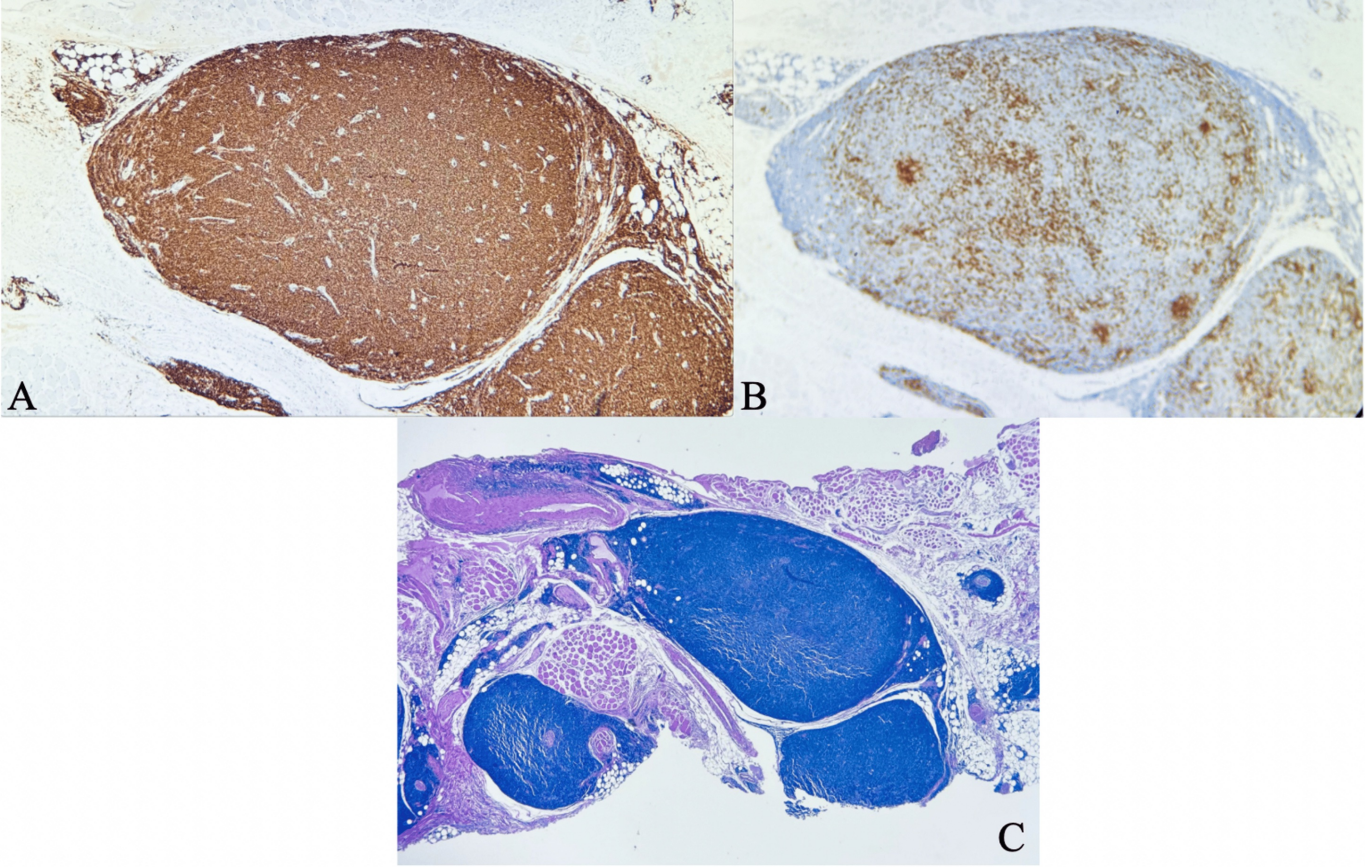
(A) Strong CD5, CD20, and CD23 immunoreactivity at 20x. (B) Staining fails to demonstrate positive immunoreactivity for the pan T-lymphocyte marker CD3 at 20x. These findings support the diagnosis of small lymphocytic lymphoma. H&E sections at 10x (C), which reveal nodular and diffuse, dermal and subcutaneous lymphoid infiltrate consistent with small lymphocytic lymphoma. There are no germinal centers or large atypical lymphoid cells.

An elderly female over the age of 90 years with a history of arthritis, asthma, and leukemia presented to an outpatient dermatology clinic for an evaluation of several new skin lesions that were present for at least two years prior to presentation. Physical examination was significant for a 1.0 cm erythematous crusted plaque located on the right superior forehead (Figure 1), which was subsequently biopsied via the shave method. Initial histopathology of the lesion showed at least SCCIS. The lesion was excised via MMS. First-stage histology revealed invasive SCC into muscle fibers with second-stage histology revealing inflammation suggestive of cancer camouflage. Third-stage histology yielded clear margins but showed dense inflammation of unknown origin with suspicion of cutaneous CLL distinct from invasive SCC. Post-Mohs histologic analysis of the stage 3 section showed nodular and diffuse, dermal and subcutaneous, lymphoid infiltrate consistent with small lymphocytic lymphoma. Subsequent immunohistochemistry revealed lymphoid cells immunoreactive for CD5, CD20, CD23, and CD43 (Figure 2). These findings suggested that the previously diagnosed SCCIS was actually CLL.

This patient did not previously carry a diagnosis of CLL, and a recommendation was made to evaluate a complete blood count with differential and peripheral blood cytometry. The patient was referred to hematology and oncology for further evaluation. 

Case 2

**Figure 3:**
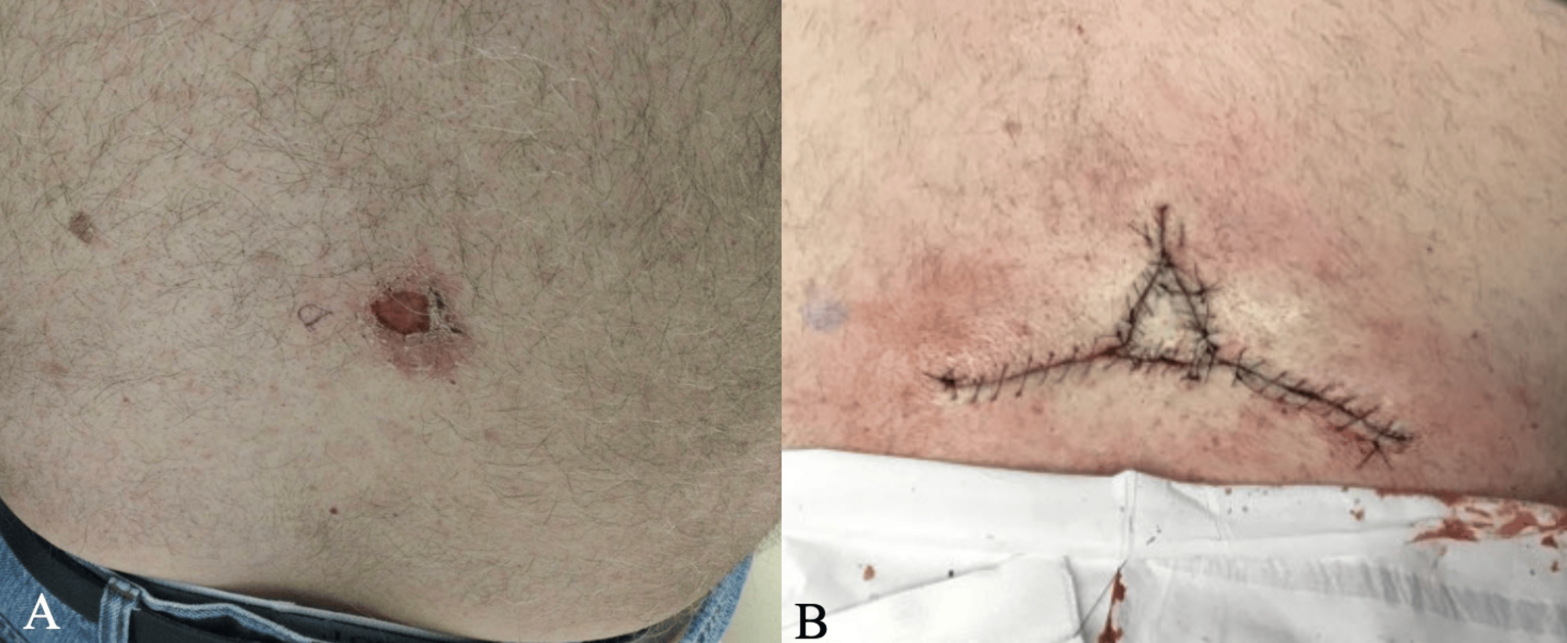
Biopsy-proven nodular basal cell carcinoma (BCC) involving the superior lumbar spine excised via Mohs micrographic surgery (MMS) (A) and repaired utilizing a Mercedes flap with subsequent V-Y flap (B).

Several months after case 1's presentation, a 73-year-old male with a medical history of hypertension and seasonal allergic rhinitis presented for definitive treatment of a 4.6 x 4.0 cm biopsy-proven nodular BCC involving the superior lumbar spine. The initial lesion was present for several years prior to presentation at the dermatology clinic for biopsy. MMS was performed on the patient. An initial debulk of the tumor was sent for permanent sections to better characterize the cell histology and depth of invasion of this large, aggressive tumor. Clear margins were attained after two stages, followed by closure utilizing a Mercedes flap with a subsequent V-Y flap (Figure 3).

**Figure 4:**
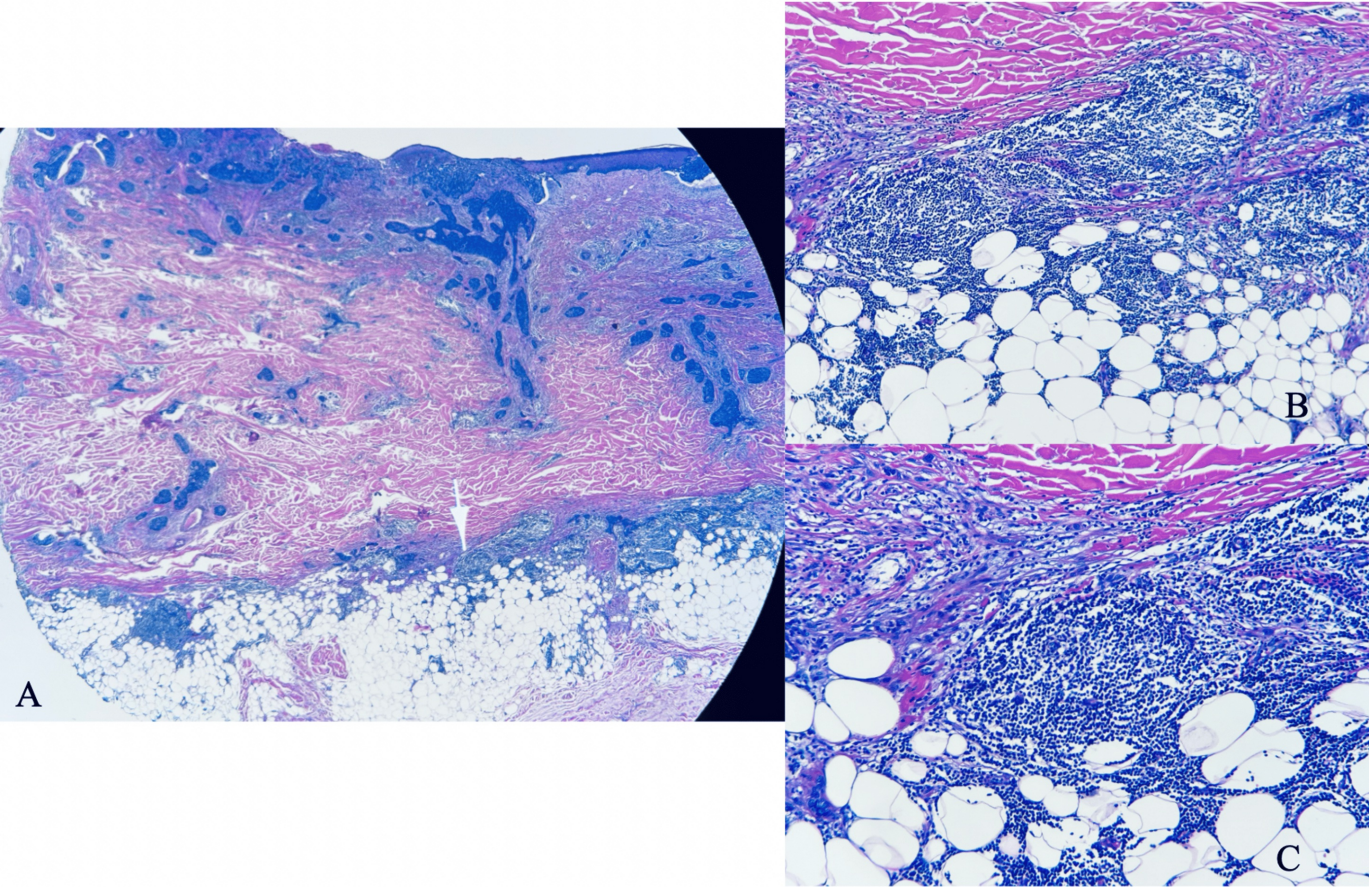
(A) Hematoxylin and eosin (H&E) stain 10x of Mohs micrographic surgery excision with the frozen section, showing extensive, residual BCC with micronodular and infiltrative features along with a diffuse and vaguely nodular infiltrate of atypical lymphoid cells within the deep dermis and subcutaneous tissue. The findings listed previously are noted on higher power, 40X magnification, in sections (B, C).

**Figure 5:**
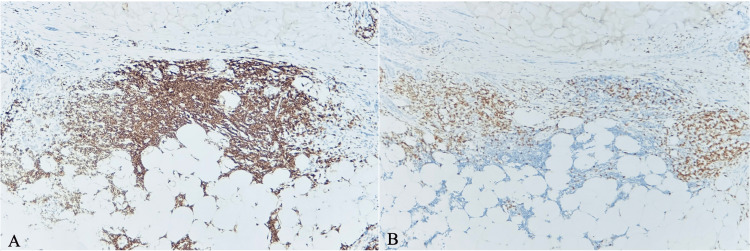
The tissue shows strong CD5, CD20, and CD23 staining (A) under high-power magnification, 40X, and fails to demonstrate positive immunoreactivity for the pan T-lymphocyte marker CD3 (B) supporting the diagnosis of chronic lymphocytic leukemia.

Histologic evaluation from the debulked tumor revealed extensive residual BCC with micronodular and infiltrative features. There was associated perineural invasion with the largest nerve diameter measuring 0.15 mm. An atypical lymphoid infiltrate with diffuse and vaguely nodular growth was present within the deep dermis and subcutaneous tissue (Figure 4). The infiltrate comprised monotonous small cells with coarsely clumped chromatin and inconspicuous nucleoli. Immunohistochemical staining revealed aberrant expression of CD5, positive CD20 and CD79a, and partially positive CD23. In the background T-cells, CD3 and CD43 were positive (Figure 5). RNA scope in-situ hybridization for kappa and lambda showed kappa restriction within the B cells and polytypic plasma cells. Ki67 showed a low proliferation index (<5%) in the lymphoma cells, and cyclin D1 was negative. This immunophenotype is compatible with CLL/small lymphocytic lymphoma diagnosis. The patient was further evaluated by oncology and did not demonstrate systemic involvement at that time.

## Discussion

CLL is the most common leukemia in Western countries, accounting for approximately 25% of all new cases of leukemia [6]. Risk factors for CLL include male sex, family history of hematologic malignancies, and people from North American and European populations [3,6]. Although primarily asymptomatic, those with symptoms may experience fevers, night sweats, weight loss, and weakness. Physical examination may reveal hepatomegaly, splenomegaly, or lymphadenopathy. The five-year survival rate of patients with CLL is 87%, with one-third of patients requiring no treatment [3,6].

Progression of CLL leads to neoplastic B lymphocyte accumulation, resulting in immune system impairment. Common complications include infectious, autoimmune, hematologic, and secondary malignancy processes. The relative risk of developing secondary cancers is 2.2, with skin cancer being among the most frequent [3,7]. The most common cutaneous neoplasms in patients with CLL are SCC and BCC, with an increased incidence of 5.0 and 2.7 compared to the normal population [8,9]. However, they do not have significantly larger BCCs or more aggressive histologic subtypes [10]. Recurrence of BCC after undergoing Mohs is 14 times higher in patients with CLL, with a cumulative incidence of recurrence of 3%, 12%, and 22% at one, three, and five years, respectively, further highlighting the importance of future monitoring [10]. 

Evaluation of a patient with suspected CLL includes a CBC with differential, peripheral blood smear analysis, and flow cytometry to assess the immunophenotypic profile. The peripheral blood smear shows numerous small mature lymphocytes with scant cytoplasm and clumped chromatin. The presence of smudge cells (Gumprecht cells), which represent ruptured cells, is a characteristic morphologic feature. The diagnostic criteria for CLL include the presence of monoclonal B lymphocytes (≥ 5 x 10⁹ /L) in the peripheral blood and an immunophenotypic profile consistent with clonal light chain restriction, CD5 expression, CD20 and 23 expression, and low levels of CD79b, and surface immunoglobulin [3,11].

Frozen section analysis on MMS presents a unique challenge to Mohs surgeons due to the common presence of an intense infiltrate surrounding the tumor [12]. Although presentation can be variable, CLL commonly presents as a more dense and monomorphic infiltrate of small mature lymphocytes with dense nuclei than is seen in reactive inflammation associated with tumor margins [13]. A characteristic feature of CLL is lymphocytes that appear flattened as a reflection of their fragility referred to as smudge cells [13]. CLL can also mimic aggressive neoplastic behavior with a persistent lymphocytic infiltrate obscuring tumor margins for multiple layers despite clearance of target neoplastic cells and even signs of perineural invasion [13]. The cases outlined in this report include one patient who exhibits CD5, CD20, CD23, and CD43 expression, while another displays CD5, CD20, CD79a, and CD23 expression. Patient presentation dictates management, but full excision with MMS is recommended followed by subsequent hematology/oncology evaluation and treatment [14].

## Conclusions

This case series highlights the importance of performing initial tumor debulking during MMS of malignant epidermal neoplasms. In both presented cases, neither patient had a known leukemia diagnosis until the histologic evaluation and immunophenotypic workup raised suspicions of CLL, prompting further testing. Heightened awareness of this overlap of skin cancer with leukemia will improve patient care in both dermatology and hematology/oncology. Molecular and cellular analysis is warranted to explore the intersection of these malignancies and further enhance our understanding of the clinical implications of co-occurring CLL and skin malignancies. Utilizing these patients' information can help prevent unnecessary complications and further establish updated guidelines for cancer diagnosis and treatment.
